# Animal modeling in bone research—Should we follow the White Rabbit?

**DOI:** 10.1002/ame2.12083

**Published:** 2019-09-26

**Authors:** Aline Schafrum Macedo, Caroline Cezaretti Feitosa, Fernando Yoiti Kitamura Kawamoto, Paulo Vinicius Tertuliano Marinho, Ísis dos Santos Dal‐Bó, Bianca Fiuza Monteiro, Leonardo Prado, Thales Bregadioli, Gabriel Antonio Covino Diamante, Cassio Ricardo Auada Ferrigno

**Affiliations:** ^1^ Department of Veterinary Surgery School of Veterinary Medicine and Animal Sciences University of São Paulo, USP São Paulo SP Brazil; ^2^ Department of Veterinary Surgery School of Agricultural and Veterinarian Sciences São Paulo State University UNESP Jaboticabal SP Brazil; ^3^ Department of Veterinary Surgery Federal Institute of Education, Science, and Technology of Southern Minas Gerais IFSULDEMINAS Muzambinho MG Brazil

**Keywords:** animal experimentation, bone density, Lagomorpha, translational medical research

## Abstract

Animal models are live subjects applied to translational research. They provide insights into human diseases and enhance biomedical knowledge. Livestock production has favored the pace of human social development over millennia. Today's society is more aware of animal welfare than past generations. The general public has marked objections to animal research and many species are falling into disuse. The search for an ideal methodology to replace animal use is on, but animal modeling still holds great importance to human health. Bone research, in particular, has unmet requirements that in vitro technologies cannot yet fully address. In that sense, standardizing novel models remains necessary and rabbits are gaining in popularity as potential bone models. Our aim here is to provide a broad overview of animal modeling and its ethical implications, followed by a narrower focus on bone research and the role rabbits are playing in the current scenario.

## 1 INTRODUCTION

In the long history of mankind, animals have played significant roles. We owe much of our intellectual sophistication and societal status to animal husbandry.^1^ Farming and agriculture have allowed humans to control their environment and an improved nutrition. The first rural settlements marked the birth of the ancient civilizations,^2^ but also triggered the first zoonotic outbreaks and the beginning of public health concerns.^3^ The earliest records of animal use coincide with the appearance of Hippocrates' concepts and the birth of Western medicine.^2^

Jumping ahead 2400 years, in the year 2018 more than 20 000 animal subjects—exceeding 200 species—were part of translational studies worldwide,^4^ in pre‐clinical trials.^2^ Animal modelling can provide reliable data to elucidate human diseases.^5^

Despite benefiting our quality of life, animal research remains controversial.^3^ There is a growing sense of disapproval over using household pets in experiments.^1^ New technologies have been created to replace animals in research, but the position of ideal bone substitute remains open.^6^ Thus, primary research on novel models continues paramount.^7^

Rabbits have been used for decades by researchers in diverse scientific fields. However, only recently have they been targeted as potential bone models.^8^ With great importance in age‐related bone loss research.^9^, ^10^ Here, we first present a broad historical review and some key ethical points in animal modeling. We then take a closer look at bone research and the role rabbits play in this field.

## 2 BACKGROUND TO ANIMAL EXPERIMENTATION

Animal domestication was a significant turning point for mankind. Human society developed into what it is today due to livestock production,^2^ and animals still provide us with food, clothing, transportation, protection, and companionship.^2^, ^11^ Nowadays they contribute to human well‐being in additional ways: by helping people with visual impairment or diabetes, by taking part in police enforcement, or even by entertaining people in animal shows, zoos, and social media.^3^

Animals have also been pivotal to our medical knowledge and health status since ancient Greece.^3^, ^9^ The first animal studies provided understanding of biological pathways and disease mechanisms. Animal dissection proved to be a valuable substitute for human dissection – an illegal practice in ancient times.^12^

Several philosophers and physicians, from Aristotle to Diocles and Erasistratus, experimented on animals. Alcmaeon of Croton (305‐240 bc) was the first physician to document and publish anatomical observations of canine dissections.^11^, ^13^ He established brain control over intelligence and sensory perceptions.^13^

Centuries later, Aelius Galenus (also known as Galen of Pergamon, 129‐216 ad) would make pivotal discoveries based on animal experimentation.^4^ Galen served as a doctor to different Roman emperors. His public demonstrations of cutting laryngeal nerves in squealing pigs made him famous. He also made important anatomic observations on cranial and spinal nerves.^14^ His theories remained undisputed until the Middle Ages.^1^, ^2^

In 1543, the Flemish physician Andreas Vesalius published his work *De Humani Corporis Fabrica*.^15^ His accurate illustrations of human anatomy diverged from Galen's ideas. Vesalius's contributions to anatomy and physiology during the Renaissance created comparative anatomy. He broke the civil and religious laws of the time by dissecting dead criminals.^2^, ^15^ A century later, William Harvey published a comparative study of the systemic circulation. He included findings from more than 80 animal species.^2^, ^16^ In the late nineteenth century, Claude Bernard set the foundations of experimental medicine by developing rigorous guidelines for controlled studies.^2^, ^4^, ^11^

Animal‐based research has been the cornerstone of health sciences ever since. It accounts for more than 80% (180/216) of all physiology or medicine Nobel Laureates’ studies.^17^ Research on the diphtheria vaccine—developed in guinea pigs (*Cavia porcellus*)—received the very first prize in 1901. Other fundamental discoveries, like the insulin mechanism and Pasteur's and Koch's studies, are also credited to animal research.^2^, ^12^, ^17^

## 3 ETHICAL CONSIDERATIONS IN ANIMAL WELFARE

Animal welfare has not always been a concern. Proper acknowledgment of an animal's moral status as a sentient being is a recent development.^2^, ^3^ For most part of the History, animals were considered senseless to pain and were treated with little or no respect in research, teaching, and demonstrations.^2^, ^12^

For centuries, animals were perceived mainly as useful tools.^2^, ^16^ Most Greek philosophers excluded animals from moral judgments, especially those derived from stoic and epicurean beliefs.^6^ Other philosophical strands, such as Cynicism, were more empathetic to the well‐being of animals. Nevertheless, assumptions that animals are entitled to ethical consideration and can indeed perceive pain and negative feelings only emerged during the Renaissance.^2^, ^11^, ^16^

French philosopher René Descartes (1596‐1650) acknowledged that animals could perceive sensations, but in a purely mechanical way. Based on this Cartesian perspective, scientists justified the use of animals without concern for their feelings for centuries afterwards.^2^, ^3^, ^6^ When William Harvey demonstrated blood circulation on conscious dogs, the attending public believed the painful screams were part of a "beast machinery," like an automatic sound.^18^, ^19^

Only by the second half of the nineteenth century, in Victorian Europe, animal rights would be debated among the mainstream philosophers.^2^, ^18^ Jeremy Bentham's *Introduction to Principles of Morals and Legislation* (1789) was a turning point.^20^ Emphatic attitudes displayed by influential thinkers like Rousseau and Schopenhauer helped shaping a new approach towards animal welfare.^3^, ^6^ Darwin's evolutionary insights (published in 1859), emphasized our moral duty towards animals.^1^, ^2^, ^3^, ^14^ The *Cruelty to Animals Act*—passed in 1876—was the first official legal document to set boundaries on animal experimentation.^21^ However, the dominant approach to animal research remained utilitarian.^2^, ^16^

In the late 1950s, Russell and Burch developed the "three Rs concept" to rationalize animal use by replacing, reducing, and refining resources.^12^ These guidelines aim to minimize animal distress and emphasize our duty to search for alternative technologies. Bioethical principles are now mandatory for any animal experimentation.^16^, ^18^

Today, the internet reflects public opinion on animal welfare. The attitude of young people towards animals is much more empathetic now than in previous generations.^2^ Consequently, bioresearch elicits heated debates. Some groups with radical views advocate banning animal research altogether. Nevertheless, the unlimited potential and importance of animal‐based discoveries cannot be denied.^12^

Five key bioethical points are considered when assessing the moral status of animal subjects in research: the presence of life, the ability to feel and perceive stimuli, the level of cognitive behavior, the degree of sociability, and the ability to proliferate.^16^ Scientific proof of animal consciousness and sentience is a recent achievement.^18^

However, there is no global consensus on the value people attach to particular animals. In some cultures, the Western household dog is no more than a food source. The same is true for research. Using animals like monkeys, dogs or cats as models will likely evoke adverse reactions nowadays. The social perception of the animal's "worthiness" is called “speciesism”.^22^

At this point in time, animal research cannot be entirely replaced by in vitro testing. Developing alternative methods is essential. Scientists can now create and cultivate microfluid organ‐on‐a‐chip models. But these new technologies are still under development. Hopefully future studies will provide the means to replace animal experiments.^23^ Until then, ethical treatment and rational use of all living forms are still necessary.^4^, ^22^

In that context, characterizing alternative models remains a goal. Rabbits, for instance, may be potentially useful bone models. They are already used as laboratory subjects in several medical fields. Even though they are also praised as household pets, particularly in Europe, their use in laboratory is well accepted.^2^, ^24^

## 4 EXPERIMENTAL MODELS

Many species can be suitable models for different diseases. The research question will dictate what type of model should be considered. Undoubtably, rodents are the most popular laboratory subjects worldwide. Rats (*Rattus norvegicus*) have been part of medical studies since the nineteenth century (1828).^25^ They reached peak importance with the development of the Wistar strain, in 1909.^2^ Although Mendel started studying the laws of inheritance on mice (*Mus musculus*), he shifted his methods to peas after facing religious restrictions on his animal model.^5^, ^6^ Rodents became the standard choice for genetic experimentation after Watson and Crick published their DNA study.^2^ During the 1980s, the first "gene knockout" mouse was developed. This study won a Nobel Prize.^2^, ^17^

Using models is very attractive because one can easily ensure homogeneity between subjects—unachievable otherwise. Then future studies can reproduce similar conditions.^11^, ^25^ For obvious reasons, the greater the model's similarity to humans, the greater are the moral implications.^6^

The planning phase is the moment to define the best model to answer the research question, avoiding unnecessary enrollments.^16^

### 4.1 The “ideal” model

The "ideal model" does not exist. No single animal—aside from humans—can perfectly exhibit human responses.^26^ Researchers must choose the most suitable option, considering the objectives of the study.^27^ Careful planning is mandatory. It should be kept in mind that sometimes more than one type of model might be necessary to answer the research question.^19^ Multi‐level assessment is required to identify the possible advantages and challenges of any given model and Table 1 provides a template guide.

**Table 1:** Schematic compilation of traits and possible challenges to consider when planning to use an animal model^2^, ^6^, ^11^

Model's trait/ challenge	Purpose/approach
Animal's kinship to humans	Defining the level of proximity to human's physiology; Assessing bioethical implications.
Genetic mechanisms; Existence of biomarkers	Setting research methods to address the objectives.
Lifespan	Defining study timeline.
Gender	Determining reproductive features.
Age	Assessing skeletal maturity.
Behavior and aggressivity level	Defining biosecurity status; Defining staff levels of expertise.
Tolerance to captivity; Ease of handling	Defining biosecurity status; Defining staff levels of expertise.
Adult body size; Activity level	Defining housing resources; Minimum space required per animal.
Zoonotic potential; Immunological features	Defining biosecurity level.
Nutritional requirements	Planning nutritional intake.
Special food; Lighting; Flooring requirement	Addressing individual needs; Defining cost budgets.
Calculating power sample	Defining the appropriate number of animals per group; Defining number of in‐house staff.
Summing up all potential costs	Defining cost budgets; assessing available funds; Applying for research grants.

### 4.2 Bone models

Animal models have taught us much about bone disorders and have been central to developing many treatments throughout history. Their contribution remains paramount for assessing bone physiology and immunology, since in vitro alternatives cannot fully reproduce whole‐organism physiological behavior. They remain beneficial to the whole orthopedic field. Either by mimicking diseases in arthrology and oncology studies or by allowing surgical training, animals are still essential to medicine.^28^

Nonhuman primates are our best biological representation.^29^ For that reason, using them nowadays for scientific purposes elicits public. Aside from moral implications, their size and ease of handling in experiments are difficulties, besides being financially demanding. Working with primates also requires very well‐trained staff (owing to their unpredictable aggressive behavior and zoonotic potential), which limits their research potential.^2^

Our second closest model on the structure of bone is dogs.^29^ Despite individual variations on macrostructure, their bone remodeling is somewhat similar, and they exhibit similar Haversian structure. Dogs used to be popular research subjects due to their medium size, ease of handling, and docile behavior.^6^, ^30^

Today, these classical models are no longer feasible.^2^, ^30^ Over recent decades, a paradigm shift regarding animal use in research has occurred. The fields of laboratory sciences, animal welfare and alternative methods for replacing animal use have expanded considerably to overcome the lack of public acceptance of the classical models.

One of the most studied—and prevalent—disorders nowadays is osteoporosis.^31^ Age‐related osteopenia is a public health concern of growing importance. Demographic aging and the urban lifestyle of Western societies have led to this modern disease. The World Health Organization considers osteoporosis a significant age‐related disease and has developed global strategies for its prevention, management, and surveillance.^32^

Osteoporosis causes unbalanced bone formation/resorption and decreases bone mass. The weakened bones are more prone to suffer a fracture, even with low‐impact injuries. Pathological fractures occur mainly at the hip joint and vertebrae. They may even go unnoticed in elder patients.^33^ These fractures severely impair the remaining self‐sufficiency of such patients, and can significantly elevate mortality rates.^7^ Secondary fractures may increase the cost of their care.^7^, ^34^, ^35^ This condition affects mainly postmenopausal women. But it can also occur, although less frequently, in elderly men.^7^

To provide accurate findings in osteoporosis studies, researchers must induce bone loss in the research subjects. This increases the complexity of the methodology and elicits further ethical issues.^36^ There are artificial methods of accelerating bone loss such as surgical procedures (ovariectomy or neutering), dietary modifications, mobility restrictions or corticosteroid administration.^37^ Animals have different estrus regimens that will interfere in osteopenia studies.^11^ An ideal animal model should display an estrogen‐related component of bone formation, more frequently encountered in polyestrous mammals.^38^

The castrated (OVX or ORX) monkey is no longer a feasible option due to the ethical implications and its unpredictable (sometimes aggressive) behavior.^6^, ^29^ The usefulness of dogs in osteoporosis research is disputable. Hormonal restriction alone does not impair bone metabolism in this monoestrous species. Genetically modified mice remain valuable in biomolecular research, even though their reduced size is a limitation.^39^

Sheep, goats, and pigs are of limited use because their bone's microstructure and remodeling processes are quite different from the human condition. In addition, the limitations imposed by the final body size and space requirements of these large animals may be challenging.^6^

## 5 RABBITS IN BONE RESEARCH: WHERE ARE WE?

The domestic rabbit (*Oryctolagus cuniculus*) is a small digging lagomorph of the family Leporidae. In the modern age, there are only two living families: Leporidae (rabbits and hares) and Ochotonidae (pikas), with 13 genera currently recognized.^40^ More than 60 rabbit breeds exist worldwide. Rabbits exhibit desirable traits for bone research. These calm and easily handled creatures have a short lifespan and breed readily in captivity.^10^

The New Zealand White Rabbit is the most popular research breed. Furthermore, rabbits are phylogenetically closer to primates than rodents. They reach skeletal maturity between 20 and 30 weeks of age (females earlier).^28^ Adults display some Haversian remodeling and their bone metabolism is somewhat similar to humans. However, surgical castration is not enough to mimic satisfactory bone loss and other techniques must be associated.^6^, ^38^

Rabbits display less cancellous bone than humans.^38^, ^41^ They have more fragile cortices.^29^, ^42^ Cortical thickness and the diameter of drilled holes contribute to the high complication rate of fracture repair in this species.^43^ Their functional anatomy allows their peculiar high‐speed hopping to evade predators. Cage confinement and exercise restriction might be harmful to their bone development^44^ and researchers should consider alternative in‐house systems, as opposed to small cage confinement.^45^

**Figure 1:**
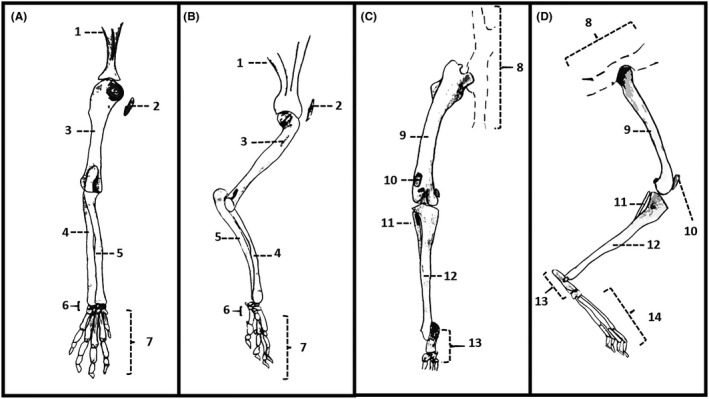
Illustration of the appendicular skeleton of the domestic rabbit (*Oryctolagus cuniculus*). A, Left forelimb, caudal view. B, Left forelimb, medial view. C, Left hindlimb, caudal view. D, Left hindlimb, medial view. 1, Scapula. 2, Clavicle. 3, Humerus. 4, Radius. 5, Ulna. 6, Carpal bones. 7, Metacarpal bones and phalanges. 8, Hemipelvis. 9, Femur. 10, Patella. 11, Fibula. 12, Tibia. 13, Tarsal bones. 14, Metatarsal bones and phalanges. Source: Aline Schafrum Macedo

In their natural habitat, rabbits are a prey species, which explains their curious but easily scared behavior and also explains some anatomic features that enable them to escape at high speed when in danger. Their peculiar appendicular skeleton (Figure 1) must be light weight but also resistant to allow their burrowing and food‐seeking behaviors.^46^ Their hindlimbs have high power hip extensor muscles concentrated at the proximal part. Muscle mass in the front limbs is distributed more distally and accounts for approximately 35% of the total body mass.^47^

Their fibula fuses to the middle shaft of the tibia. Their four long webbed toes on each hindlimb allow accelerated digitigrade hopping. Their small clavicles resemble those of domestic cats and make them more agile.^48^, ^49^

A survey of the terms "rabbit" and "experimental model" in PubMed resulted in 33 344 articles of indexed journals published between 1951 and 2019, with almost 10 000 from the past decade. Rabbits were pivotal to the discovery of the atropine esterase enzyme, in the nineteenth century.^50^ Since then, they have been used in several studies by Nobel Laureates. They helped characterizing the mechanisms involved in insulin production and diabetes.^8^, ^17^, ^51^

Rabbits are appealing models for bone research. Studies involving rabbits are now commonplace in orthopedics, and multi‐species assessments of model suitability have rated rabbits as potential bone models after primates and dogs.^52^ Biomechanical forces act during stance and walking in any living animal. Measuring these forces is important to determine a model's bone strength,^53^ but biomechanical data on rabbit bones are still scarce.

A 2012 study published the effects of in vivo loading of rabbit tibiae. Biomechanical data on axial compression and bending moments in the rabbit tibia were given. The authors concluded that rabbit tibia can endure higher strain levels than goats can, therefore rabbits were better models.^54^ In another study describing the qualitative differences between mice, rats, dogs, nonhuman primates and rabbits, the authors concluded that the skeletal characteristics of rabbits were the least suitable for extrapolating to humans, but highlighted their lack of biomechanical data.^52^

Rabbits are a standard model in periodontal research. They are part of diverse studies such as measurement of parathyroid hormone effects on osseointegration in osteoporosis,^55^ measurement of bone healing of a zinc‐containing nanostructured porous hydroxyapatite scaffold,^56^ assessments of varied biomaterials like hydroxyapatite combinations,^57^, ^58^ and bioceramics.^59^

Some recent studies have explored the potential of rabbits as models for cartilage^60^ and meniscal tears repair.^61^ They have also been used in other studies on arthrology and tendon healing. One study focused on intra‐articular injections of chondroitin sulfate carried by hydrogel.^62^ Others assessed tendon healing by reproducing biceps tenosynovitis,^63^ and anterior cruciate ligament^64^ and rotator cuff tears.^65^

Rabbits have also increased in importance as pets. They are the third most popular companion animal in the UK, after dogs and cats. More than two million pet rabbits are estimated to have existed in the past decade.^66^ They are the most popular exotic animal in US private veterinary practices.^24^ In view of these trends, the demand for higher standards of rabbit medicine is increasing and thus the need to enhance veterinary knowledge also exists.^24^

More recent studies focus on clinical and surgical aspects of the pet rabbit.^24^, ^43^, ^67^, ^68^, ^69^, ^70^, ^71^, ^72^ In a recent paper, the authors evaluated the effect of three different screw‐hole diameters and torsional properties of rabbit femora.^43^ However, more in‐depth biomechanical studies are lacking. There are scarce data on the torsional properties,^73^, ^74^, ^75^ but the main focus of these studies was bone healing^74^ and bone grafting.^75^

Fracture repair in the pet rabbit remains a major challenge.^68^ Rabbit bones are very thin and brittle, an important complicating factor that results in frequent implant failure.^43^, ^76^ Another study has defined vertebral safe corridors for implant insertion using computer tomography.^77^ But rabbit research still has unexplored gaps to be addressed.

## 6 SUMMARY AND FINAL CONSIDERATIONS

The human‐animal bond has sculpted medical knowledge. Animal models play a significant role in enhancing our understanding of emerging pathologies. Current in vitro technologies are very promising but still have some way to go before fully replicating whole‐animal responses. Rabbits have potential as bone models but conclusive studies are still lacking. However, the growing popularity of rabbits as pets may ultimately decrease their eligibility as laboratory models. The need for alternative methods to replace animals in research remains paramount.

## CONFLICT OF INTEREST

None.

## AUTHOR CONTRIBUTIONS

ASM, PVTM and CRAF conceived the review. ASM, CCF and FYKK conducted the literature research, analyzed all the relevant data and wrote the manuscript. The other authors helped during the literature research, corrected the manuscript several times and provided insights for the final version. All the authors read, contributed and agree with the content of the final version of the manuscript.
